# Primary ocular adnexal mantle cell lymphoma with distant spread and involvement of the contralateral eye one year later; a case report and literature review

**DOI:** 10.1093/jscr/rjae414

**Published:** 2024-06-11

**Authors:** Amaar Amir, Baraa Amir, Salwa Sheikh

**Affiliations:** Imam Abdulrahman Bin Faisal University, College of Medicine, Dammam 31441, Saudi Arabia; Imam Abdulrahman Bin Faisal University, College of Medicine, Dammam 31441, Saudi Arabia; Pathology Services, Pathology Services, John Hopkins Aramco Healthcare, Dhahran 34455, Saudi Arabia

**Keywords:** ocular adnexal lymphoma, mantle cell lymphoma, eyelid, conjunctiva

## Abstract

We herein report a middle-aged gentleman who initially presented with ocular adnexal mantle cell lymphoma (MCL) on the right eyelid. The lesion was excised and the patient was treated with radiation therapy. During the initial presentation, a PET CT was performed and did not reveal disease involvement beyond the eyelid. The patient presented 3 months later with ocular adnexal MCL of the contralateral eye. Re-evaluation using PET CT revealed a slight increase in the uptake in several lymph nodes and the spleen, which, after biopsy, confirmed systemic MCL. The patient was started on six cycles of chemotherapy. The patient also underwent autologous hematopoietic stem cell transplant. Approximately 80% of primary ocular adnexal lymphomas are B-cell in origin, with MCL being the rarest subtype constituting only 5% of B-cell ocular adnexal lymphomas. Despite its rarity, it is crucial for clinicians to detect the entity early and ensure rapid initiation of appropriate therapy.

## Introduction

Lymphoma of the ocular adnexa, which comprises the tissue surrounding the eye and optic nerve, is a rarely encountered entity. Of the ocular adnexal lymphomas, mantle cell lymphoma (MCL) is the least frequently encountered. We herein report a case of a middle-aged gentleman who presented with a lesion on the right eyelid that was excised and histopathologically confirmed as ocular adnexal MCL. The patient was treated and presented 3 months later with ocular adnexal MCL of the contralateral eye after complaining from a suspicious mass on the left conjunctiva.

### Case presentation

A 58-year-old male patient presented to the ophthalmology clinic after noticing a small lesion on the medial aspect of the right eyelid. The patient noted progressive increase in the size of the lesion over the course of a few months. History of fever, chills, night sweats, and significant weight loss were denied. The patient’s past medical history was positive for class 3 obesity, diabetes mellitus type 2, hypertension, hyperlipidemia, chronic obstructive pulmonary disease, liver cirrhosis, thrombocytopenia, severe non-proliferative diabetic retinopathy with macular edema in right eye, and right eye cataract status post cataract extraction by phacoemulsification with posterior chamber intraocular lens placement. The patient’s surgical history was also positive for a carpal tunnel release and removal of a tubular adenoma from the ascending colon.

**Figure 1:**
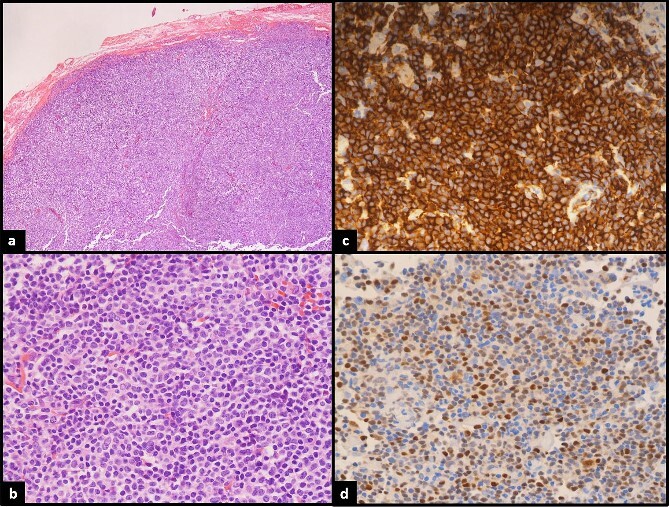
(a)Low power: neoplastic lymphoid proliferation showing vague nodular architecture. (b) High power: atypical lymphocytes are medium in size with mostly round uniform nuclei and smooth nuclear membranes. (c) CD20 strongly positive by most of the lymphoma cells confirming B lineage. (d) Cyclin-D1 positive in the lymphoma cells.

An excisional biopsy was performed of the right upper eyelid. Histopathological examination revealed a well circumscribed mass that displayed a dense and diffuse lymphoid infiltrate of small to medium sized cells with scant cytoplasm, irregular nuclear contours, and condensed chromatin. Immunoperoxidase studies were performed and revealed that the neoplastic cells are positive for CD5, CD20, CD43, BCL2, Cyclin D1, IgD, with Ki-67 proliferation of 10–15%. Interpretation of the kappa and lambda immunoglobulin light chain expression revealed lambda light chain restriction (Fig. 1). A provisional diagnosis of atypical lymphoproliferative changes was made; however, upon review, a final diagnosis of MCL was provided. The patient underwent a PET CT scan which at the time did not reveal any definitive evidence of disease beyond the right eyelid. As such, the patient was referred to radiation oncology for radiation therapy where a total dose of 2400 cGy was successfully delivered in 12 fractions to the entire right eye and ocular adnexa.

During a routine ophthalmology follow-up approximately 3 months later, a small lesion in the left conjunctiva was noted and biopsied. Histopathological examination revealed similar morphology to the previously excised eyelid lesion with findings supporting the diagnosis of MCL. Re-evaluation using PET CT revealed slight increase in uptake in several lymph nodes and the spleen. The largest area of enhancement was in the left inguinal area. A biopsy from the inguinal region confirmed the presence of MCL. The patient at this point was complaining of a fever and persistent cough. Whilst the fever resolved, the cough remained, and a follow-up chest X-ray showed a right upper lobe infiltrate. Bronchoscopy was performed and showed oedematous mucosa of the trachea and carina. There was a nodule in the right main bronchus. Another nodule was present in the posterior segment of the right upper lobe bronchus which was surrounded by areas of increased vascularity. Biopsy of this lesion showed MCL. The patient was started on six cycles of Rituximab, Cyclophosphamide, Doxorubicin, Vincristine, and Prednisone. The patient also underwent autologous hematopoietic stem cell transplant. The first cycle of chemotherapy was tolerated well by the patient; complete remission workup is pending after the sixth cycle.

## Discussion

Lymphomas involving the eyelids, conjunctiva, lacrimal drainage system, and orbital contents excluding the globe and optic nerve are categorized as ocular adnexal lymphomas (OAL) [1]. The most commonly involved sites are the conjunctiva and orbit whereas involvement of the lacrimal drainage system is considered rare [2]. OALs are further categorized into primary if only the ocular adnexa is involved and secondary if there is evidence of systemic lymphoma of the same type prior to or at the time of diagnosis of the ocular lesion. Approximately one-third of patients presenting with primary OALs will develop systemic lymphoma in the 10 years following diagnosis of the ocular lesion. Conversely, only 5% of patients with systemic Non-Hodgkin’s lymphoma (NHL) will develop OALs during their disease course [3]. Primary OALs represent 1–2% of all NHL and 5–15% of all extranodal NHLs [4]. The majority of primary OALs are B-cell in origin, whereas 20% of OALs are reported to arise from T-cells and natural killer cells with the former comprising 14% and the latter comprising 6% [3]. An international multicenter retrospective study conducted in 2019 analysing orbital lymphoma cases across a 24-year period found that the most common primary OALs are extranodal marginal zone B-cell lymphoma (EMZL) at 59% of cases, followed by diffuse large B-cell lymphoma (DLBCL) and follicular lymphoma (FL) at 23 and 9%, respectively. The least common type of OAL reported was MCL at only 5% [5]. Though percentages may vary across the literature, these percentages are concordant with most studies regarding OALs [6].

**Table 1:** Summary of current and prior case reports describing primary ocular adnexal mantle cell lymphoma

Patient gender	Patient age	Chief complaint at presentation	Anatomic location of lesion	Positive immunohistochemistry	Ann Arbor stage	Administered treatment	Disease status at last visit	Reference
M	64	Bilateral exophthalmos, palpebral ptosis, reduced visual acuity	Retroorbital region bilaterally	CD5, CD20, CD22, Cyclin D1	IV	RCHOP, RDHAP, intrathecal Methotrexate and Cytarabine, BEAM, HCT	Complete remission	[15]
M	70	Epiphoria, bilateral rubbery painful salmon-coloured conjunctival masses	Bilateral inferior conjunctiva	CD20, BCL2, Cyclin D1, Ki-67 at 15–20%	IV	CVP	Receiving treatment	[16]
M	58	Incidental detection of conjunctival mass	Left lower tarsal conjunctiva	CD20, BCL2, Cyclin D1, Ki-67 15%	IV	CVP	Receiving treatment	[17]
M	78	bilateral progressively enlarging rubbery painful salmon-coloured eyelid masses	Bilateral superior temporal aspect of anterior orbits	CD5, CD20, CD43, BCL2, Cyclin D1, Ki-67 at 20–30%	IV	N/A	N/A	[12]
M	64	Dark red palpable mass in right bulbar conjunctiva	Bulbar conjunctiva at medial canthus of right eye	CD5, CD20, BCL2, Cyclin D1, Ki-67 at 12%	IV	FCR	Partial remission	[18]
F	61	Progressively enlarging painless, salmon-coloured conjunctival mass	Right nasal conjunctiva	CD5, CD20, BCL2, Cyclin D1, Ki-67 at 50%	IV	Bendamustine and Rituximab	Complete remission	[19]
M	52	Redness of left eye with excessive lacrimation	Left temporal bulbar conjunctiva	CD20, Cyclin D1	I	RCVP	Receiving treatment	[20]
M	55	Swelling left supraorbital ridge and upper eyelid	Intraconal and extraconal compartments of left orbit	CD20, Cyclin D1	I	RCHOP	Partial remission	[20]
M	77	Painless progressive left globe proptosis, progressively enlarging left upper lid margin lesion, left sided tearing	Left intraconal and extraconal compartments of superior orbit	PAX5, Cyclin D1	IV	Rituxan and Lenalidomide, Abdominal radiotherapy 400 cGy	Receiving treatment	[21]
M	61	Bilateral conjunctival erythema, watery discharge, proptosis	Bilateral superior lateral anterior orbits	CD5, CD19, CD20	IV	R-CHOP, BEAM, autologous HCT	Complete remission	[22]
M	71	Progressive ptosis of right eye	Intraconal compartment of right orbit	CD20, CD5, Cyclin D1	I	Orbital radiotherapy 36 Gy	Complete remission	[23]
M	78	Bilateral painless, palpable, red, conjunctival masses	Right inferior temporal conjunctiva and left superior nasal conjunctiva	CD20, BCL2, SOX11, Cyclin D1, Ki-67 at 6–10%	I	Bendamustine and Rituximab	Died from pneumonia following chemotherapy	[24]
M	58	Progressively enlarging mass on eyelid	Medial aspect of right upper eyelid	CD5, CD20, CD43, BCL2, Cyclin D1, IgD, Ki-67 at 10–15%	I	Orbital radiotherapy 24 Gy, RCHOP, HCT	Receiving treatment	Current study

M, male; F, female; R-CHOP, Rituximab, Cyclophosphamide, Doxorubicin, Vincristine, Prednisone; RDHAP, Rituximab, Dexamethasone, High dose AraC Platinol; RCVP, Rituximab, Cyclophosphamide, Vincristine, and Prednisolone; CVP, Cytoxan, Vincristine, Prednisone; BEAM, Carmustine, Etopaside, Cytarabine, Melphalan; FCR, Fludarabine, Cyclophosphamide, Rituximab; HCT, hematopoietic cell transplant

Rasmussen et al. demonstrated that MCL of the ocular adnexa typically occurs in older patients with a median reported age of 75 years. Males are at a higher risk of developing the condition in comparison to females with a reported 6:1 male to female ratio that far exceeded that of systemic MCL [7]. This is in contrast to EMZL and FL which generally show a female predominance [5]. Primary MCL was more likely to occur than secondary MCL, and typically involved the orbits and eyelids. The duration of symptoms prior to consultation with an ophthalmologist varies significantly between different subtypes of OALs. Patients with ocular adnexal MCL typically present late with a median duration of symptoms of 9 months reported. This is considerably longer than the few weeks or months prior to presentation in patients with more aggressive subtypes such as DLBCL and FL. Patients presenting typically complain of ptosis, proptosis, diplopia, change in visual acuity, salmon-pink lesions, swelling, restriction of ocular movement, and conjunctival erythema. Patients presenting with secondary ocular adnexal MCL may exhibit signs of lymphadenopathy, splenomegaly, and pancytopenia. Whilst the overall prognosis of patients with ocular adnexal MCL is generally poor compared to other forms of lymphoma, patients presenting with primary lesions have a shorter survival rate than those with secondary lesions [7]. Most OALs of B-cell origin present unilaterally; however, varying studies report patients with ocular adnexal MCL are more likely to exhibit bilateral involvement than other subtypes [5, 7]. On cytological examination, MCL can be subdivided into four types, namely small cell, marginal zone like, pleomorphic, and blastoid.

Our literature review yielded 12 case reports describing primary ocular adnexal MCL not including the case discussed in the current study. Of the reported cases, only a single patient was a female. The mean age was 72 years with the lowest age reported being 52 years and the highest reported being 78 years. In eight of the reported cases, the chief complaint was a recently noticed swelling or mass. The remaining cases reported other presenting complaints including conjunctival erythema, exophthalmos, ptosis, excessive tearing, and reduced visual acuity. Five cases reported bilateral involvement on presentation. Immunohistochemistry findings suggested that the majority of cases reported were positive for CD5 (6 cases), CD20 (11 cases), and Cyclin D1 (11 cases). According to the Ann Arbor Staging criteria, eight cases were stage IV on presentation, whereas the remaining four cases were stage I. Treatment regimens varied significantly in the reported cases, and ranged from external beam radiotherapy (EBRT) alone in localized disease to the incorporation of chemotherapy for systemic disease. Hematopoietic cell transplant is frequently offered as part of the treatment algorithm. Of the reported cases, four achieved complete remission, six were still receiving treatment or in partial remission, one patient passed away from pneumonia following chemotherapy, and one case did not report information on the outcome of the patient. The patient discussed in the current study presented with signs and symptoms that were concordant with the cohort mentioned in Table 1 with the exception of the fact that the patient initially had a unilateral lesion that later evolved to include systemic involvement and involvement of the contralateral eye. Additionally, presenting with stage 1 disease seemed to be less common in the cases discussed. Otherwise, the lesion was CD5, CD20, and Cyclin D1 positive, and the patient is doing well after receiving chemotherapy, EBRT, and HCT.

MCL is typically associated with translocation t(11; 14)(q13;q32), which leads to deregulation of Cyclin D1 by juxtaposing the BCL-1 locus to the immunoglobulin gene sequence [8, 9]. While a hallmark of the disease, Cyclin D1 positivity also requires a host of secondary chromosomal abnormalities in order to cause overt MCL. Gain chromosomal abnormalities have been reported in 3q, 7q, and 8q, while chromosomal abnormalities that include losses have been reported in 1p, 6q, 8p, 9p, 9q, 11q, 13q, and 17p [10, 11]. Of note is that Cyclin D1 as a marker may present a potential diagnostic pitfall as certain MCL cases may not possess this marker, which is unusual as the presence of Cyclin D1 is typically associated with t(11; 14), one of the trademark features of MCL [12]. Similarly, there may exist other forms of lymphoma that exhibit Cyclin D1 positivity. In such rare cases, this form of MCL may present with high expression of Cyclin D2, Cyclin D3, or SOX11 [13, 14]. It is recommended to use fluorescence *in situ* hybridization to distinguish these forms of MCL from other lymphomas [12].

## Conclusion

Herein, we report an exceedingly rare case of a middle-aged gentleman who initially presented with eyelid swelling later confirmed as primary ocular adnexal lymphoma of the right eye, only to receive treatment and present once more several months later with ocular adnexal MCL of the contralateral eye which manifested as a conjunctival mass. Cases of primary ocular adnexal MCL are few and far between and have seldom been reported in detail. There is still much to discover in relation to the underlying genetic and cytochemical makeup of the disease, as well as the optimal treatment of the disease. Awareness of this rare presentation of ocular adnexal MCL by clinicians and pathologists is of utmost importance to aid in correctly diagnosing and treating such patients.

## Data Availability

The data used to support the findings of this study are included within the article.
